# Role of clinical laboratories in response to the COVID-19 pandemic

**DOI:** 10.4155/fmc-2020-0129

**Published:** 2020-06-19

**Authors:** Nikhil Shri Sahajpal, Allan Njau, Ashis K Mondal, Sudha Ananth, Alka Chaubey, Amyn Rojiani, Ravindra Kolhe

**Affiliations:** ^1^Department of Pathology, Medical College of Georgia, Augusta University, GA 30912, USA; ^2^Department of Pathology, Aga Khan University Hospital, Nairobi, Kenya; ^3^Global Laboratory Services, PerkinElmer, Waltham, MA 02451, USA

The outbreak of COVID-19 (caused by SARS-CoV-2) is now a pandemic that has caused a global socio-economic disorder. Since its identification in the region of Wuhan, China, 2,629,579 confirmed cases with over 182,900 COVID-19 related deaths have been reported globally [1]. In response to the outbreak, several state authorities and commercial companies have developed diagnostic assays to test individuals for the SARS-CoV-2 infection. Currently, over 40 diagnostic assays have received Emergency Use Authorization (EUA) from the Federal US FDA for COVID-19 testing. In the US, clinical laboratories are required to perform ‘bridging studies’ on FDA approved SARS-CoV-2 diagnostic assays to implement testing under the EUA regulation.

Absence of adequate testing due to various factors, most significant of which being supply chain issues, is most likely contributing to community spread. In midst of numerous challenges, clinical laboratories have a critical role to play in response to the current COVID-19 pandemic. In addition to ensuring the testing requirements of the population in the present hour, laboratories have an unprecedented responsibility to prepare for the aftermath of the pandemic. Although, the reverse transcription-polymerase chain reaction (RT-PCR) based assays for the detection of SARS-CoV-2 nucleic acid regions might be the most practical approach at present, qualitative assays are far from providing insights into the evolution of the virus and the varied immune response in different populations. Herein, we discuss the three main categories of diagnostic assays available for the identification of SARS-CoV-2 infection, their utility and a way-around the challenges associated with each assay. Further, laboratory management issues are highlighted that might be considered by laboratories for optimal functioning.

## Nucleic acid detection assays

The nucleic acid detection assays have two primary components, first: RNA extraction from clinical specimen and second: RT-PCR based detection of SARS-CoV-2 nucleic acid region(s). The nucleic acid targets are based on primer/probe sequences published by either USA or China CDC, targeting selected regions of the virus nucleocapsid (N), envelop (E) or open reading frame genes. The panels target multiple regions in the same gene, or multiple genes, in addition to an internal control to monitor assay performance. The current challenge facing diagnostic laboratories using RT-PCR based assays is deficits in supply, impeding efforts to ramp up testing. Sample collection has also been hampered due to lack of viral transport media. Although EUAs are accompanied by recommendations of the ideal test protocol, laboratories have to increase COVID-19 test output without compromising on accuracy yet with less than ideal variables. To eliminate testing constrains, we optimized various facets of SARS-CoV-2 detection assay, ranging across pre-analytical and analytical laboratory variables. The pre-analytical constraints emerged as the viral transport media used for collecting nasopharyngeal (NSP) swab samples (most common sample type) became exhausted, forcing laboratories to hold up sample collection, or revert to other collection methods (in different media or sample types). To validate the alternate transport media and sample types, we performed ‘bridging studies’ as per FDA recommendations, using three serial dilutions of the SARS-CoV-2 viral material in universal transport media (UTM), viral transport media (VTM), 0.9% NaCl, Amies media and broncho-alveolar lavage (BAL) samples, which demonstrated comparable results with these modifications. In addition, 3D print swabs were validated as a sample collection tool by comparing NSP and 3D print swab data from 20 patients. The validation of BAL samples helped us to screen ICU patients on ventilators, as NSP samples could not be collected from this sub-group of patients. Further, as the test kits are in short supply, we maximized our testing potential by optimizing the RNA extraction and RT-PCR reaction with minimum reagent input. However, the sensitivity of the RT-PCR instrument must be considered while optimizing the reaction volume. Overall, we have observed that various sample types such as NSP and BAL, collected using conventional NSP swabs, e-swab or 3D printed swabs and, preserved in VTM, UTM, NaCl or Amies media are compatible with RT-PCR assay for COVID-19.

In addition, the RT-PCR based assays provide a unique opportunity to implement pooling sample strategy for wide-scale population screening for SARS-CoV-2. Pooling samples compared with individual testing has been used previously, such as in screening blood donations, infectious and genetic diseases. Several studies, including from our laboratory (under review) have demonstrated that pooling sample strategy is a practical and feasible method for screening populations for SARS-CoV-2 [2]. An important consideration is to optimize the number of samples to be pooled based on the incidence rate of the region where the testing is being performed. The approach has the potential to maximize screening, with minimum turnaround time and utilization of resources.

## Serology assays

Serological assays that detect SARS-CoV-2 IgA and IgM have also entered into the fray of COVID-19 pandemic control. Although peak viral loads are seen in the first few days of infection [3], seroconversion and therefore antibody detection rates, occurred maximally in the second week of infection [4]. This negatively impacts the sensitivity of serology in the early phase of infection but serves an important role later on in the course of the disease as viral loads decline. Another point of consideration is cross reactivity that has been observed especially with SARS-CoV-2. When factors affecting its clinical performance are dully considered, serology has demonstrated utility when paired with PCR resulting in higher detection rates compared with PCR alone (98.6 vs 51.9%). Positive identification of subclinical patients who were negative for RT-PCR by ELISA for IgM has also been documented [5]. Serologic assays are more easily performed and have a short turnaround time compared with RT-PCR. They are also highly scalable to be adopted for mass screening especially in the exposed but asymptomatic population. Laboratories should therefore prime for serologic testing by validating assays using RT-PCR confirmed COVID-19 samples. In addition to screening potential blood donations and convalescent plasma donors, serology might be an important piece in the puzzle of triaging individuals who may be susceptible from those who are potentially immune and not actively shedding virus. Evaluation of this assay on serum as well as dry blood spots on an automated ELISA system would be best suited to minimize the variability of manual assays.

## Next-generation sequencing

Laboratories approved for high complexity testing such as Clinical Laboratory Improvement Amendments labs are also in a position to explore next-generation sequencing (NGS) as a potential test for coronaviruses. Given that in just less than two decades, three coronavirus outbreaks have occurred; SARS in 2002, MERS in 2012 and the still active SARS-CoV-2, the probability for further outbreaks is likely. The capacity for unbiased identification of genomes, positions NGS as a critical tool for identification of novel infectious agents that may facilitate early containment of outbreaks [6]. Several studies have also demonstrated its utility in monitoring viral evolution [7]. Although currently limited by considerations of cost, improvements in the technology and multiplexing may see it being adopted for clinical use, as has happened in clinical oncology and other infectious diseases.

## Laboratory management issues

In addition to total quality management surrounding pre-analytic, analytic and post-analytic processes, several key aspects of laboratory management will ensure smooth running of laboratory operations. Active follow-up of : efficient and timely procurement of all supplies needed; consistent compliance to laboratory safety manuals/guidelines regarding all hazards; space management to ensure tests are performed without contamination and with an efficient workflow; optimized storage of reagents vis à vis ensuring adequate stock; archival of SARS-CoV-2 specimens within regional/institutional guidelines; effective and accurate record keeping and billing. With increased work load, these aspects can be overwhelming or easily forgotten, therefore, laboratories should have practical checklists to guide operations within available resources. Application of quality improvement principles such as Six Sigma and Lean Management principles, may be useful.

## Take home message

Laboratories should adopt a multi-pronged strategy in assay development, that are cost effective, accurate, time efficient and that cater for mass testing, differing clinical scenarios, uninterrupted or sustainable testing in case of supply chain failures and enhance further research and understanding of COVID-19. Due attention to laboratory management will facilitate smooth operations. Currently, resources to equip laboratories have been awarded or increased to meet the need for COVID-19 testing. A careful consideration of an effective COVID-19 testing program, plus a look into how these resources can be redefined for improved testing beyond COVID-19 and/or a better preparedness for future outbreaks is needed.
